# Supplementary material for: Biosynthesis of Arabinoside from Sucrose and Nucleobase via a Novel Multi-Enzymatic Cascade
Source: Biomolecules. 2024 Sep 3;14(9):1107. doi: 10.3390/biom14091107 (PMC11430244; doi:10.3390/biom14091107)
Supplement: Supplementary file 1 [file biomolecules-14-01107-s001.zip › SI for arabinoside from sucrose.pdf]

Supporting Information

Biosynthesis of arabinoside from sucrose and nucleobase via a novel multi-enzymatic cascade

Yuxue Liu, Erchu Yang, Xiaojing Zhang, Xiaobei Liu, Xiaoting Tang, Zhenyu Wang*, Hailei Wang*

Henan Engineering Research Center of Bioconversion Technology of Functional Microbes, College of Life Science, Henan Normal University, Xinxiang 453007, China. liuyuxue@htu.edu.cn (Y.L.); yangerchu@126.com (E.Y.); 1970411725@qq.com (X.Z.); 2993304817@qq.com (X.L.); 1316329782@qq.com (X.T.)

* Corresponding author. E-mail address: wangzhenyu@htu.cn (Z. Wang); whl@htu.cn (H. Wang).

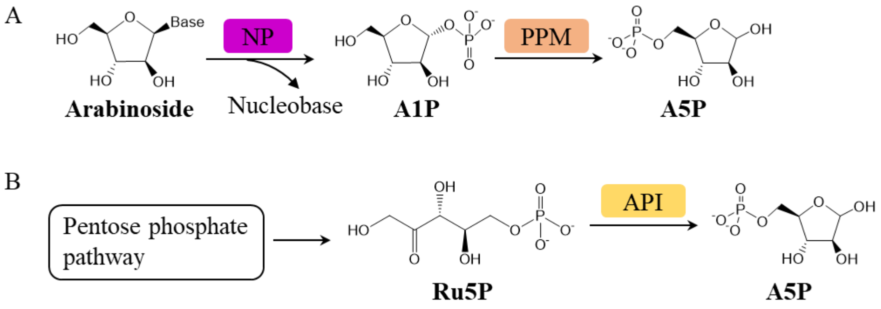

Figure S1. Bacterial nucleoside salvage pathway applied to arabinosides.

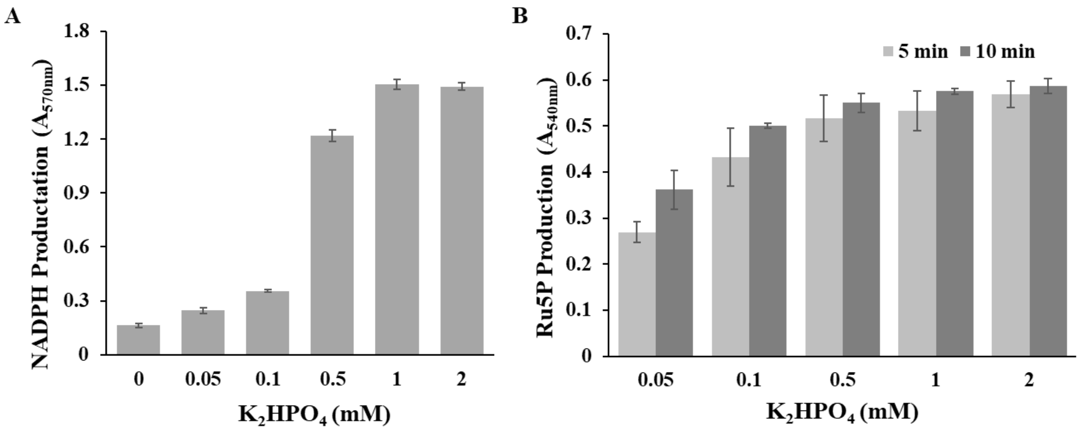

Figure S2. Effect of phosphate concentrations on the accumulation of intermediate metabolite. A, Accumulation of NADPH with the 6-phosphogluconate production from sucrose. The reaction mixture contains 10 µM *Ao*SP, 10 µM *Ec*PGM, 10 µM *Ec*G6PD, 0.5 mM sucrose, 2 mM $MgCl_2$, 0.02 mM $NADP^+$, 0.4 mM MTT and 1 mM PES. After adding phosphate, the reactions were incubated at room temperature for 1 h. NADPH was determined by monitoring the absorbance at 570 nm. B, Ru5P production from sucrose. The reaction mixture contains 10 µM *Ao*SP, 10 µM *Ec*PGM, 10 µM *Ec*G6PD, 5 µM *Ec*6PGDH, 0.5 mM sucrose, 2 mM $MgCl_2$, and 2 mM $NADP^+$. After adding phosphate, the reactions were incubated at room temperature for 1 h.

Table S1. The plasmids and strains used in this study.

Strain or plasmid	Characteristic	Source
Strain		
DH5*α*	F⁻ φ80*lac*ZΔM15 Δ(*lacZYA-argF*) U169 *deoR recA1 endA1 hsdR17*(rK–, mK+) *phoA supE44 λ– thi-1 gyrA96 relA1*	Lab collection
BL21(DE3)	F⁻ *ompT hsdSB* (rB- mB-) *gal* (*λ c I 857 ind1 sam7 nin5 lacUV5-T7gene1*) *dcm* (DE3)	Lab collection
Plasmid		
pET28a	Vector for protein expression; Kanr	Lab collection
pET28a-*Ec*G6PD	pET28a with *zwf* gene from *E. coli*	This study
pET28a-*Ec*6PGDH	pET28a with *gnd* gene from *E. coli*	This study
pET28a-*Ec*API	pET28a with *kdsD* gene from *E. coli*	This study
pET28a-*Bc*PPM	pET28a with codon optimized *deoB* gene from *Bacillus cereus*	This study
pET28a-*Ao*SP	pET28a with codon optimized *gtfA* gene from *Alloscardovia omnicolens*	This study
pET28a-*Ec*PGM	pET28a with *pgm* gene from *E. coli*	This study
pET28a-TPNOX	pET28a with codon optimized TPNOX gene from *Lactobacillus brevis*	This study
pET28a-*Kl*PNP	pET28a with *deoD* gene from *Klebsiella*	Lab collection
pET28a-*Ec*PNP	pET28a with *deoD* gene from *E. coli*	Lab collection
pET28a-*Ec*UP	pET28a with *udp* gene from *E. coli*	Lab collection
pET28a-*Aa*PNP	pET28a with *deoD* gene from *Alicyclobacillus acidoterrestris*	Lab collection
pET28a-*Hs*RK	For the expression of ribokinase isoform 1 from *Homo sapiens*	Lab collection

Table S2. The primers used in this study.

Oligonucleotide	Sequence (5′ to 3′)
pET28a-F	ctcgagcaccaccaccac
pET28a-R	CATggtatatctccttc
zwf-F	AAGAAGGAGATATACCATGgcggtaacgcaaacagccc
zwf-R	TGGTGGTGGTGGTGGTGctcgagctcaaactcattccagg
gnd-F	gaaggagatataccATGTCCAAGCAACAGATCG
gnd-R	gtggtggtggtgctcgagATCCAGCCATTCGGTATGG
kdsD-F	gaaggagatataccATGTCGCACGTAGAGTTACAACC
kdsD-R	gtggtggtggtgctcgagCACTACGCCTGCACGCAGTAAATCATG
pgm-F	AAGGAGATATACCATGgcaatccacaatcgtgcaggcc
pgm-R	ATCTCAGTGGTGGTGGTGGTGGTGctcgagcgcgtttttcagaacttc
