# Supplementary figures and images for: Biosynthesis of Arabinoside from Sucrose and Nucleobase via a Novel Multi-Enzymatic Cascade
Source: Biomolecules. 2024 Sep 3;14(9):1107. doi: 10.3390/biom14091107 (PMC11430244; doi:10.3390/biom14091107)

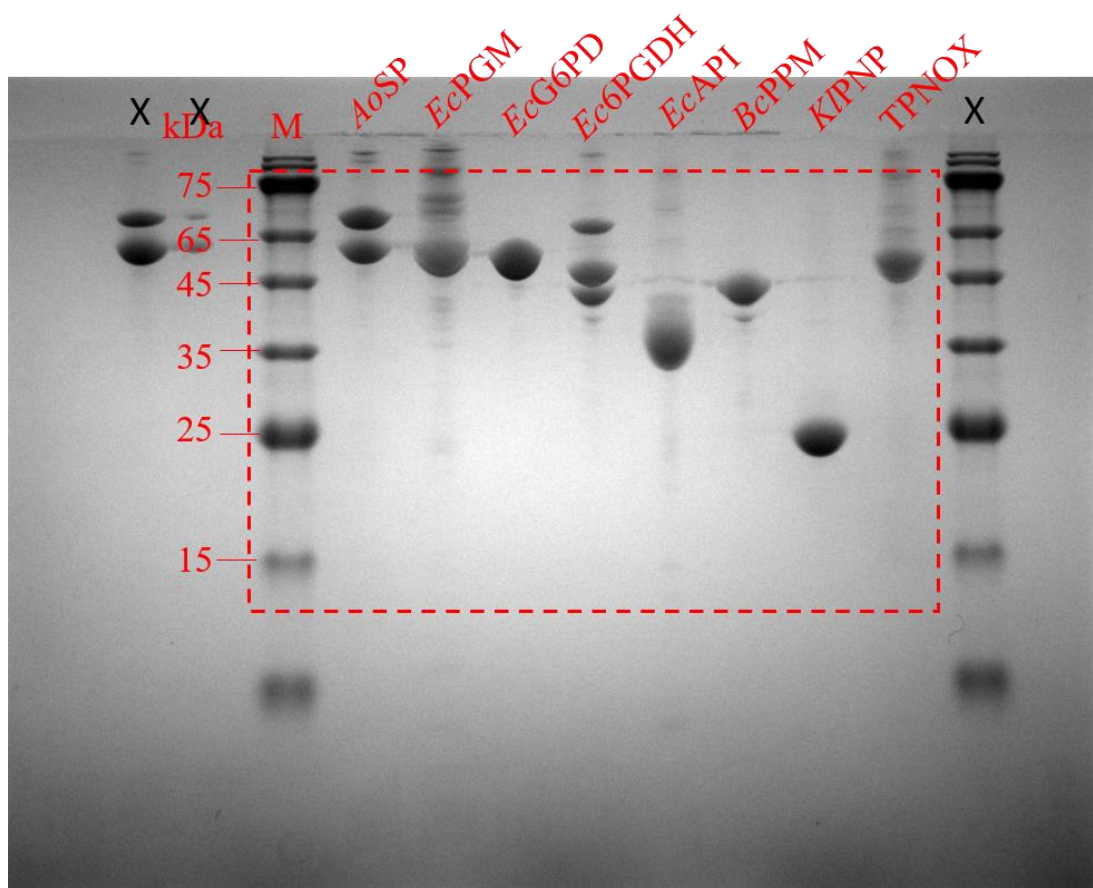

The original gel image corresponding to Figure 2B

Supplement: Supplementary file 1 [file biomolecules-14-01107-s001.zip › biomolecules-3169670-original-images.pdf]
